# Hypolipidemic and Antiobesity-Like Activity of Standardised Extract of *Hypericum perforatum* L. in Rats

**DOI:** 10.5402/2011/505247

**Published:** 2011-04-12

**Authors:** Gulam Mohammed Husain, Shyam Sunder Chatterjee, Paras Nath Singh, Vikas Kumar

**Affiliations:** ^1^Pharmacology Research Laboratory, Department of Pharmaceutics, Institute of Technology, Banaras Hindu University, Varanasi 221 005, India; ^2^Pharmacology Research Laboratories, Dr. Willmar Schwabe GmbH & Co. KG, Karlsruhe, Germany; ^3^Stettiner Straße 1, 76138 Karlsruhe, Germany

## Abstract

*Hypericum perforatum* is known to have diverse medicinal uses for centuries. The antidepressant activity of *Hypericum perforatum* is widely accepted and proved in both animal and clinical studies. Present study was undertaken to investigate the effect of *Hypericum perforatum* in a battery of animal models for metabolic disorder. *Hypericum* is tested for hypolipidemic activity in normal rats, antiobesity activity in high-fat-diet induced obese rats, and fructose-fed rats. *Hypericum* was orally administered as suspension in 0.3% carboxymethyl cellulose at the doses of 100 and 200 mg/kg body weight for 15 consecutive days. *Hypericum* significantly lowered total cholesterol and low-density cholesterol in normal rats. *Hypericum* significantly inhibited weight gain in high-fat-fed rats. In fructose-fed rats, *Hypericum* normalised the dyslipidemia induced by fructose feeding and improved the insulin sensitivity. Taken together, *Hypericum* could be the antidepressant therapy of choice for patients suffering from comorbid diabetes and obesity.

## 1. Introduction

*Hypericum perforatum *L. (Family: *Clusiaceae*), also called St. John's wort, is widely distributed in Europe, Asia, North Africa, and North America. In India, *Hypericum perforatum *is found in the western Himalayas at altitudes of 3000–10,500 feet [1]. *Hypericum perforatum* is widely used as complementary and alternative medicine by patients suffering from a range of CNS disorders [2–7]. The antidepressant potential of *Hypericum* is widely accepted. It is now well established that prolonged treatment with synthetic antidepressant drugs markedly increased the risk of weight gain and obesity [8]. Moreover, synthetic antidepressant drugs are also reported to increase the risk of development of type 2 diabetes [9]. Recently, *Hypericum perforatum* extract and hyperforin, a major bioactive constituent of *Hypericum perforatum*, have been reported to protect cytokine-induced *β*-cell injury, thereby improving *β*-cell function and survival [10] which could be potentially valuable for the prevention or limitation of beta-cell loss, observed in diabetes. A couple of studies from our laboratory found a significant antihyperglycemic activity of *Hypericum perforatum *extract in diabetic rats [11, 12]. Therefore, we propose that *Hypericum* could be the antidepressant therapy of choice for patients suffering from comorbid diabetes or obesity. In view of putative antiobesity activity, *Hypericum perforatum *will gain a new perspective as an antidepressant therapy.

Animal models are useful tools for obesity research as they readily gain weight when fed with high-fat diets [13]. The rats fed with high fat develop obesity, hyperphagia, hyperleptinemia, hyperinsulinemia, hyperglycemia, and hypertriglyceridemia [14]. The physiological aspects of this model replicate many of the features observed with the human obesity syndrome [15]. Therefore, the high fat fed model has a good translation potential to extrapolate animal data for clinical studies. Rats, maintained on high-fructose diet, develop an acute hypertriglyceridemia and insulin resistance [16–18]. Fructose is more lipogenic than glucose or starch and induces moderate obesity and several adverse metabolic effects, including hypertriglyceridemia, hyperinsulinemia, and hypertension in rodents [19]. Fructose bypasses the phosphofructokinase regulatory step and enters the pathway of glycolysis or gluconeogenesis at the triose phosphate level, resulting in increased hepatic triglyceride production [20] and insulin resistance [21]. The abnormalities and the disease progression in fructose-fed rats resemble the human condition of metabolic syndromei hence, this model also has good predictive validity. In the present communication, hypolipidemic activity of *Hypericum perforatum* was assessed in normal rats, and two validated models were used to assess the antiobesity activity, that is high-fat-fed model and fructose-fed model.

## 2. Materials and Methods

### 2.1. Animals

Adult Charles Foster rats (150 ± 10 g) were obtained from the Central Animal House of Institute of Medical Sciences, Banaras Hindu University, Varanasi, India. The animals were housed in groups of six in polypropylene cages at an ambient temperature of 25 ± 1°C and 45–55% relative humidity, with a 12 : 12 h light/dark cycle. Animals were provided with commercial food pellets and water *ad libitum*, except if otherwise stated. All the animals were acclimatized to laboratory conditions for at least one week before using them for the experiments. Principles of laboratory animal care guidelines (NIH publication number 85–23, revised 1985) were followed. Prior approval from the Institutional Animal Ethics Committee was obtained (Letter no. Dean/2009-10/693).

### 2.2. Plant Extract

Dried hydroalcoholic (50%) extract of whole plant of *Hypericum perforatum *L. was used in the present study. Standardised extract was procured from Indian Herbs Research and Supply Co. Ltd., Saharanpur, India. The extract of *Hypericum perforatum *(HpE) was standardised by HPLC to contain not less than 3.00% hyperforin and 0.3% hypericinsi thus, the extract used in the present study was similar to the extracts of *Hypericum* previously studied in our laboratory [2, 4–6].

### 2.3. Administration of Plant Extract

HpE was suspended in 0.3% carboxymethyl cellulose (CMC) and administered orally through orogastric tube at the doses of 100 and 200 mg/kg of body weight per day for 15 consecutive days. Doses are selected based on the earlier studies from our laboratory on the same extract [2, 4–6, 11].

### 2.4. Hypolipidemic Activity in Normal Rats

Rats were maintained on normal pellet diet (NPD) and orally treated with HpE 100 and 200 mg/kg for a period of 15 days. Control group was treated with CMC throughout the 15 days of study. Clofibrate 100 mg/kg served as standard hypolipidemic drug and was orally administered for 15 days [18]. Blood samples were collected on the 15th day after appropriate fasting under ether anaesthesia, for the estimation of total cholesterol (TC), HDL cholesterol (HDL-C), LDL cholesterol (LDL-C), and triglyceride (TG) as in our earlier study [12]. Total cholesterol estimation was based on the hydrolysis of cholesterol esters by cholesterol esterase to free cholesterol and fatty acids. The free cholesterol was then oxidized by cholesterol oxidase to cholest-4-en-3-one with the simultaneous production of hydrogen peroxide. The hydrogen peroxide produced was coupled with 4-aminoantipyrine and phenol, in the presence of peroxidase, to yield a chromogen with maximum absorbance at 505 nm. The absorbance of coloured dye was proportional to the total cholesterol concentration present in the sample. For HDL cholesterol estimation, LDL cholesterol, VLDL cholesterol and chylomicron fractions were precipitated by the addition of polyethylene glycol 6000. After centrifugation, the HDL fraction remained in the supernatant and was analysed in the same manner as mentioned in the total cholesterol estimation. For triglycerides estimation, triglycerides were hydrolysed by lipoprotein lipase to produce glycerol and free fatty acid. In the presence of glycerol kinase and adenosine triphosphate, glycerol was phosphorylated to glycerol-3-phsophate and adenosine diphosphate. Glycerol-3-phosphate was further oxidised by glycerol-3-phosphate oxidase to yield dihydroxyacetone phosphate and H_2_O_2_. H_2_O_2_ was then coupled with 4-aminoantipyrine and 4-chlorophenol in the presence of peroxidase to produce red quinoneimine dye. The absorbance of coloured dye was measured at 505 nm and was proportional to triglycerides concentration present in the sample. LDL cholesterol was calculated using Friedewald's equation [22].

### 2.5. Fructose-Induced Hypertriglyceridemia and Insulin Resistance

Rats were maintained on normal pellet diet (NPD) and 20% fructose in drinking water for 15 days [16–18]. Control rats were given NPD and normal drinking water throughout the study period. Rats were randomly divided into different groups as follows:

Group-I: NPD + normal drinking water + 0.3% CMC (16th to 30th day),
Group-II: NPD + 20% fructose water + 0.3% CMC (16th to 30th day),
Group-III: NPD + normal drinking water + 0.3% HpE 100 mg/kg (16th to 30th day),
Group-IV: NPD + normal drinking water + HpE 200 mg/kg (16th to 30th day),
Group-V: NPD + 20% fructose water + HpE 100 mg/kg (16th to 30th day),
Group-VI: NPD + 20% fructose water + HpE 200 mg/kg (16th to 30th day).

The administration of herbal extract was started from the 16th day and was continued up to the 30th day of experiment. Group-I (normal control) and Group-II (fructose control) rats were given equal volume of vehicle (0.3% CMC suspension) for the same duration. The body weight of rats was recorded on the 1st day and subsequently once a week, likewise food and water intake was also recorded on a weekly basis [18]. Blood samples were withdrawn from retroorbital venous plexus on the 30th day after appropriate fasting, and plasma TC, HDL-C, LDL-C, TG, and glucose were estimated using biochemical kits. Glucose was estimated by glucose oxidase/peroxidase method as in our earlier studies [11, 12]. Briefly, glucose was converted to gluconic acid and H_2_O_2_ in the presence of glucose oxidase. Subsequently, in a peroxidase-catalysed reaction, the oxygen liberated was accepted by the chromogen system to give a red-coloured quinine-imine compound. The absorbance of red colour was measured at 505 nm and was directly proportional to glucose concentration. Plasma insulin level was estimated using enzyme-linked immunosorbent assay (ELISA kit; DRG Diagnostics, GmbH, Germany).

### 2.6. High-Fat-Diet-Induced Obesity

Rats were maintained on an NPD for one week before the start of experiment. After one week, rats were randomly assigned into normal and obese groups and fed with NPD and HFD, respectively, for 15 days. High-fat diet was made as described by Srinivasan et al. [23]. After 15 days, the HFD-fed rats showing significant weight gain compared to the NPD rats were again divided into three groups (six rats in each group): the HFD control group fed with HFD only, the HFD + HpE (100 mg/kg) group, and the HFD + HpE (200 mg/kg) group. HpE treatment was started from the 16th day up to day 30. Control group rats were provided with NPD for the entire 30 days of study. Control group and HFD control group were given equal volume of 0.3% CMC suspension from the 16th to the 30th day. 

The body weight was recorded on day one and then on a weekly basis. Average food intake was recorded on the 1st, 15th, and the 30th day. Fasting blood samples were collected on 30th day from the retro-orbital venous plexus under light ether anesthesia. Plasma glucose, insulin, TC, LDL-C, HDL-C, and TG level were estimated. On the 30th day, after the collection of blood sample, animals were sacrificed and mesenteric, perirenal, and epididymal fat pads were isolated and weighed [24].

### 2.7. Statistical Analysis

Data was expressed as mean ± standard error of mean (SEM) for each group (*n* = 6). Statistical analysis was performed by one-way analysis of variance (ANOVA) followed by the Student-Newman-Keuls test. GraphPad InStat (version 3.06) software was used for statistical analysis.

## 3. Results

### 3.1. Hypolipidemic Activity in Rats

15 days of oral administration with HpE resulted in significant decrease of plasma total cholesterol (*F*(3,20) = 19.69;  *P* < .001) and LDL-C level compared to vehicle-treated control group. Clofibrate treatment decreased total cholesterol, LDL-C (*F*(3,20) = 18.56; *P* < .001), and triglyceride with simultaneous increase in HDL-C compared to control group (*F*(3,20) = 10.37; *P* < .001). Results are summarised in Table 1. There was no statistically significant difference in body weight of different treatment group throughout the study (data not shown).

### 3.2. Fructose-Induced Hypertriglyceridemia and Insulin Resistance in Rats

#### 3.2.1. Blood Glucose and Insulin Level in Fructose-Fed Rats

HpE administration in rats maintained on normal pellet diet and normal drinking water did not significantly change plasma glucose or insulin level compared to vehicle-treated normal control rats. Fructose-fed rats showed a marked increase in plasma glucose level along with a significant increase in plasma insulin level compared to vehicle-treated normal control rats. Both doses of HpE significantly inhibited the increase in plasma glucose caused by fructose feeding (*F*(5,30) = 54.11; *P* < .001). HpE 200 mg/kg significantly decreased plasma insulin level compared to fructose-fed control group (*F*(5,30) = 60.39; *P* < .001). Results are presented in Table 2.

#### 3.2.2. Effect of HpE on Lipid Parameters in Fructose-Fed Rats

Fructose feeding to rats resulted in impairment in normal lipid profile leading to increased total cholesterol, LDL-C, and triglyceride level while HDL-C was decreased. Triglyceride level of fructose-fed rats increased up to 3 times higher than vehicle-treated normal rats. HpE dose dependently and significantly decreased total cholesterol (*F*(3,20) = 13.05; *P* < .01) and TG level (*F*(3,20) = 77.06; *P* < .001) while HDL-C was increased compared to vehicle-treated fructose fed rats (*F*(3,20) = 10.66; *P* < .01). Results are summarized in Table 3.

#### 3.2.3. Effect of HpE on Body Weight in Fructose-Fed Rats

Fructose-fed rats significantly gained weight compared to normal rats (*F*(5,30) = 8.18; *P* < .001). The oral administration of HpE for 15 days did not alter the body weight of rats compared to vehicle-treated control rats. HpE administration reduced body weight gain induced by fructose feeding; however, data remained statistically insignificant (Table 2).

### 3.3. High-Fat-Diet-Induced Obesity

#### 3.3.1. Effect of HpE on Body Weight and Food Intake in HFD-Induced Obese Rats

**Figure 1:**
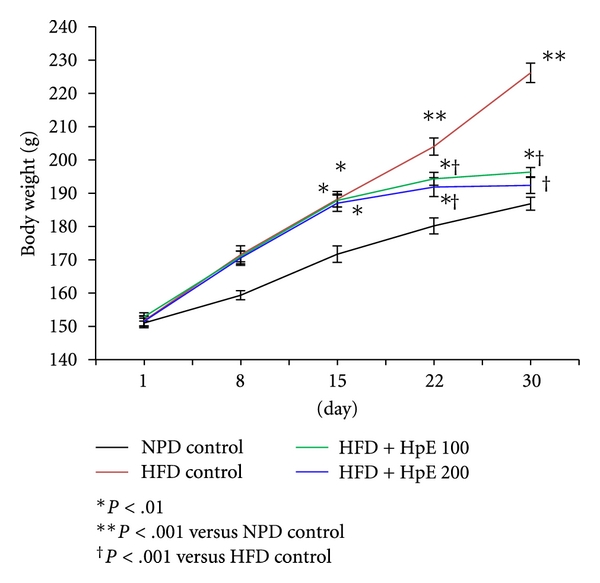
Effect of *Hypericum perforatum *on body weight changes in high-fat-induced obese rats.

**Figure 2:**
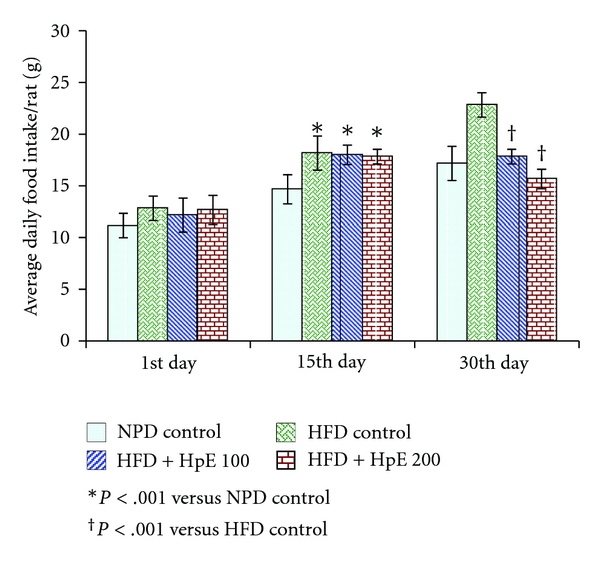
Effect of *Hypericum perforatum *on average daily food intake in high-fat-induced obese rats.

There was no significant difference in body weight of different treatment groups at the commencement of study. Animals fed with high-fat diet showed significant increase in body weight compared to those fed with NPD. HpE dose dependently and significantly inhibited the increase in body weight induced by high-fat diet (*F*(3,20) = 61.21; *P* < .001). On day 30, there was no statistically significant difference in body weight of 200 mg/kg HpE-treated HFD-fed rats and NPD control rats (Figure 1). The average daily food intake of all the groups was the same at the commencement of study; however, 15 days feeding with high-fat diet significantly increased the average food intake on the 15th day. Treatment with HpE significantly reduced the food intake on day 30 compared to the HFD control group (*F*(3,20) = 25.97; *P* < .001). Results are depicted in Figure 2.

#### 3.3.2. Effect of HpE on Lipid Parameters in HFD-Induced Obese Rats

Animals of HFD control groups showed a significant increase in plasma total cholesterol, LDL-C, and triglyceride level while plasma HDL-C was significantly decreased compared to NPD control animals. Both doses of HpE significantly reduced TC (*F*(3,20) = 37.51; *P* < .001), LDL-C (*F*(3,20) = 55.68; *P* < .001), and TG (*F*(3,20) = 37.50; *P* < .01) level while HDL-C was increased significantly (*F*(3,20) = 22.33; *P* < .05). Results are presented in Table 4.

#### 3.3.3. Effect of HpE on Blood Glucose and Insulin in HFD-Induced Obese Rats

HFD fed-rats showed a moderate but significant increase in plasma glucose level (average plasma glucose level = 130 mg/dL) a long with a significant increase in plasma insulin level compared to vehicle-treated normal control rats. HpE dose dependently and significantly inhibited the increase in plasma glucose caused by high-fat diet (*F*(3,20) = 34.01; *P* < .001) along with a simultaneous decrease in plasma insulin level (*F*(3,20) = 88.02; *P* < .001) compared to vehicle-treated HFD control group (Table 5).

#### 3.3.4. Effect of HpE on Adipose Tissue in HFD-Induced Obese Rats

**Figure 3:**
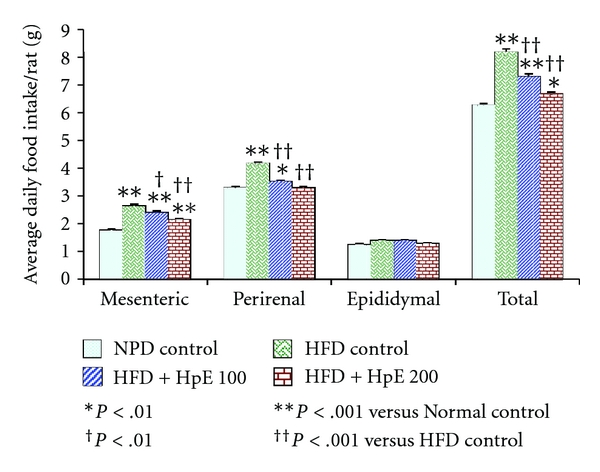
Effect of *Hypericum perforatum* on various adipose tissues in high-fat-induced obese rats.

Figure 3 demonstrates the effect of various treatments on mesenteric, perirenal, and epididymal fat pads. There was a significant increase in total adipose tissue in HFD control group compared to NPD control rats. HpE significantly inhibited the accumulation of body fat in mesenteric and perirenal regions compared to vehicle-treated HFD control rats (*F*(3,20) = 76.78; *P* < .001). However, total adipose tissue contents of all HFD groups were significantly higher than NPD control rats.

## 4. Discussion

In our study, 15 days of repeated oral administration of *Hypericum perforatum* extract and clofibrate have shown a significant hypolipidemic activity in normal rats. Elevated plasma lipoprotein level, especially hypercholesterolemia, results from increased absorption of cholesterol from the intestine or enhanced endogenous synthesis [25]. Therefore, there could be two possible underlying mechanisms of observed hypolipidemic activity of plant extracts, that is, the blockade of biosynthesis of cholesterol or decrease in dietary cholesterol absorption from the intestine by binding with bile acids within the intestine and increasing bile acids excretion. 

In the present study, HpE not only showed hypolipidemic activity in normal rats but also normalised the lipid abnormalities induced by HFD or fructose feeding. Effects of HpE on lipid parameters are consonant with the earlier finding of Laggner et al. [26]. HpE also significantly reduced the increased plasma glucose level in fructose or HFD-fed rats indicating improvement in insulin functions. Thus, reversal of fructose-induced insulin resistance appears to be the likely mechanism responsible for the observed effects of HpE on lipid parameters.

HpE showed significant inhibition in weight gain induced by high-fat diet or fructose feeding. Serotonin is important neurotransmitter that controls increase in body mass and is involved in the pathophysiology of obesity as well as depression [27]. Serotonin release is increased upon intake of carbohydrates. Serotonin regulates the overconsumption of carbohydrate-rich foods. Serotonin has also been reported to decrease food intake in fructose-fed rats [28]. *Hypericum perforatum *increases the quantity of serotonin present within synaptosomes by inhibiting synaptosomal uptake of serotonin [29]. This increased level of serotonin caused by HpE reduces the food intake and suppresses the appetite [30, 31]. Thus increased serotonergic transmission might be the connecting link between antidepressant- and antiobesity-like activity of HpE.

## 5. Conclusion

The present study demonstrates that HpE decreases body weight gain, serum parameters (total cholesterol, LDL-C, triglyceride, glucose, insulin) and increases HDL-C in fructose and HFD-fed rats. Taken together, *Hypericum* could be the antidepressant therapy of choice for patients suffering from comorbid diabetes and obesity.

##  Conflict of Interests

Authors declare no conflict of interests in the present work.

## Figures and Tables

**Table 1:** Effect on *Hypericum perforatum *and clofibrate on plasma lipid parameters in normal rats.

Group (*n* = 6)	Total-C (mg/dL)	HDL-C (mg/dL)	LDL-C (mg/dL)	Triglyceride (mg/dL)
Control (CMC)	83.98 ± 1.34	31.89 ± 1.21	41.46 ± 2.21	53.10 ± 2.49
HpE 100 mg/kg	73.29 ± 2.10**	35.24 ± 1.56	29.02 ± 2.73*	45.11 ± 2.70
HpE 200 mg/kg	70.51 ± 1.61**	36.64 ± 1.42	25.20 ± 1.60**	43.33 ± 3.67
Clofibrate 100 mg/kg	64.96 ± 2.04**	42.19 ± 1.09**	15.17 ± 3.25**	38.00 ± 2.14*

*P < .01,  **P < .001 versus Control group.

**Table 2:** Effect of *Hypericum perforatum *on plasma glucose, insulin, and body weight gain in fructose-fed rats.

Group (*n* = 6)	Glucose (mg/dL)	Plasma insulin (*μ*IU/mL)	Body weight gain (g)
Normal control (normal drinking water + CMC)	80.52 ± 3.44	18.05 ± 0.79	12.33 ± 1.05
Fructose control (fructose + CMC)	149.66 ± 4.49**	35.03 ± 1.24**	26.83 ± 2.59**
Normal drinking water + HpE 100 mg/kg	82.53 ± 3.35^ ††^	19.37± 0.95^††^	11.50 ± 1.48^††^
Normal drinking water + HpE 200 mg/kg	78.18 ± 1.71^††^	20.29 ± 0.78^††^	11.83 ± 1.30^††^
Fructose + HpE 100 mg/kg	125.44 ± 5.70^∗∗ †^	32.32 ± 1.10**	21.00 ± 3.62*
Fructose + HpE 200 mg/kg	105.15 ± 3.88^∗∗ ††^	30.58 ± 0.83^∗∗ †^	20.17 ± 2.17*

*P < .05,  **P < .001 versus normal control; ^†^ *P* < .01,  ^††^ *P* < .001 versus fructose control.

**Table 3:** Effect on *Hypericum perforatum *on plasma lipid parameters in fructose-fed rats.

Group (*n* = 6)	Total-C (mg/dL)	HDL-C (mg/dL)	LDL-C (mg/dL)	Triglyceride (mg/dL)
Normal control (normal drinking water + CMC)	81.68 ± 2.36	36.21 ± 1.41	35.98 ± 1.65	47.48 ± 3.15
Fructose control (fructose + CMC)	117.12 ± 5.12***	27.38 ± 1.35***	62.04 ± 4.89**	138.52 ± 4.88***
Fructose + HpE 100 mg/kg	106.01 ± 4.35**	30.12 ± 0.92**	57.25 ± 5.57**	93.24 ± 4.12^∗∗∗††^
Fructose + HpE 200 mg/kg	96.99 ± 4.25^∗†^	33.69 ± 1.01^†^	47.21 ± 4.84	80.50 ± 4.80^∗∗∗††^

*P < .05,  **P < .01,  ***P < .001 versus normal control; ^†^ *P* < .01,  ^††^ *P* < .001 versus fructose control.

**Table 4:** Effect of *Hypericum perforatum *on plasma lipid parameters in high-fat-induced obese rats.

Group (*n* = 6)	Total-C (mg/dL)	HDL-C (mg/dL)	LDL-C (mg/dL)	Triglyceride (mg/dL)
Normal control (NPD + CMC)	53.60 ± 2.27	34.65 ± 0.92	33.55 ± 3.07	53.60 ± 2.27
HFD control (HFD + CMC)	105.03 ± 4.87**	24.62 ± 0.79**	116.49 ± 6.68**	105.03 ± 4.87**
HFD + HpE 100 mg/kg	86.27 ± 2.51^∗∗††^	28.24 ± 0.89^∗∗†^	80.72 ± 3.83^∗∗†††^	86.27 ± 2.51^∗∗††^
HFD + HpE 200 mg/kg	80.42 ± 3.61^∗∗†††^	30.19 ± 0.93^∗†††^	62.85 ± 4.21^∗∗†††^	80.40 ± 3.61^∗∗†††^

*P < .01,  **P < .001 versus normal control; ^†^ *P* < .05,  ^††^ *P* < .01,  ^†††^ *P* < .001 versus HFD control.

**Table 5:** Effect of *Hypericum perforatum *on plasma glucose and insulin in high-fat-induced obese rats.

Group (*n* = 6)	Glucose (mg/dL)	Plasma insulin (*μ*IU/mL)
Normal control (NPD + CMC)	84.96 ± 3.58	16.61 ± 0.86
HFD control (HFD + CMC)	130.89 ± 3.70**	62.14 ± 2.95**
HFD + HpE 100 mg/kg	112.15 ± 3.08^∗∗†^	36.47 ± 2.25^∗∗†^
HFD + HpE 200 mg/kg	98.89 ± 3.01^∗†^	28.78 ± 1.53^∗∗†^

*P < .01,  **P < .001 versus normal control; ^†^ *P* < .001 versus HFD control.
